# Ureter Smooth Muscle Cell Orientation in Rat Is Predominantly Longitudinal

**DOI:** 10.1371/journal.pone.0086207

**Published:** 2014-01-21

**Authors:** Bart Spronck, Jort J. Merken, Koen D. Reesink, Wilco Kroon, Tammo Delhaas

**Affiliations:** 1 Department of Biomedical Engineering, CARIM School for Cardiovascular Diseases, Maastricht University, Maastricht, The Netherlands; 2 Institute of Computational Science, University of Lugano, Lugano, Switzerland; National Cancer Center, Japan

## Abstract

In ureter peristalsis, the orientation of the contracting smooth muscle cells is essential, yet current descriptions of orientation and composition of the smooth muscle layer in human as well as in rat ureter are inconsistent. The present study aims to improve quantification of smooth muscle orientation in rat ureters as a basis for mechanistic understanding of peristalsis. A crucial step in our approach is to use two-photon laser scanning microscopy and image analysis providing objective, quantitative data on smooth muscle cell orientation in intact ureters, avoiding the usual sectioning artifacts. In 36 rat ureter segments, originating from a proximal, middle or distal site and from a left or right ureter, we found close to the adventitia a well-defined longitudinal smooth muscle orientation. Towards the lamina propria, the orientation gradually became slightly more disperse, yet the main orientation remained longitudinal. We conclude that smooth muscle cell orientation in rat ureter is predominantly longitudinal, though the orientation gradually becomes more disperse towards the proprial side. These findings do *not* support identification of separate layers. The observed longitudinal orientation suggests that smooth muscle contraction would rather cause local shortening of the ureter, than cause luminal constriction. However, the net-like connective tissue of the ureter wall may translate local longitudinal shortening into co-local luminal constriction, facilitating peristalsis. Our quantitative, minimally invasive approach is a crucial step towards more mechanistic insight into ureter peristalsis, and may also be used to study smooth muscle cell orientation in other tube-like structures like gut and blood vessels.

## Introduction

Contraction and relaxation of smooth muscle cells (SMCs) of the ureter are responsible for active propulsion of urine from the kidneys to the bladder by peristalsis. Although it is accepted that orientation of SMCs plays an important role in peristalsis [1], morphological reports on the lamina muscularis (LM) are inconsistent. Findings with respect to the number of layers that are distinguishable by orientation and with respect to the specific orientation of SMCs within the layers (circumferential, longitudinal, helical, disperse) differ between studies. For example, a number of investigators reported different layers in the LM of human ureters based on distinct SMC orientation patterns [2]–[9], whereas others did not find a well-defined layering in SMC orientations [10]–[19]. Moreover, some of the investigators found longitudinal and/or circumferential orientations [2]–[8], [16], while others observed helical/interwoven SMC orientations [9], [15], [17], [20]. In rat ureter, similar discrepancies are found, i.e., some authors describe the rat ureter's LM as being layered with an outer longitudinal and an inner circular layer [21]–[23], whereas other groups describe no clear layering [10], [24], or an inner longitudinal and outer circular layer [25]. It is important to note that methodological aspects may play a role in these discrepancies. Most studies evaluated the LM's structure by histological sectioning methods, which may affect tissue morphology and provides limited capabilities for quantifying SMC orientation across the thickness of the LM.

The aim of the present study was to develop a quantitative approach to study SMC orientation 1: in intact ureters at approximate *in vivo* geometry, 2: throughout the entire thickness of the LM, 3: with sufficient (depth) resolution, and 4: with consideration of potential differences along the length of the ureter or between left and right ureters. Given that in rat ureter similar discrepancies in terms of SMC stratification and within-layer orientation as in human were found, we used whole ureters from wild type rats as a model. In order to avoid artifacts due to histological fixation and sectioning, we used two-photon laser scanning microscopy (TPLSM) to image intact ureters that were mounted between glass pipettes at approximate *in vivo* length and diameter. The adequate penetration depth of TPLSM allowed us to transverse the entire LM from out- to inside.

## Concise Materials and Methods

### Ethics statement

Experiments and procedures were approved by the Maastricht University animal experiments committee.

### Experimental procedures

**Figure 1:**
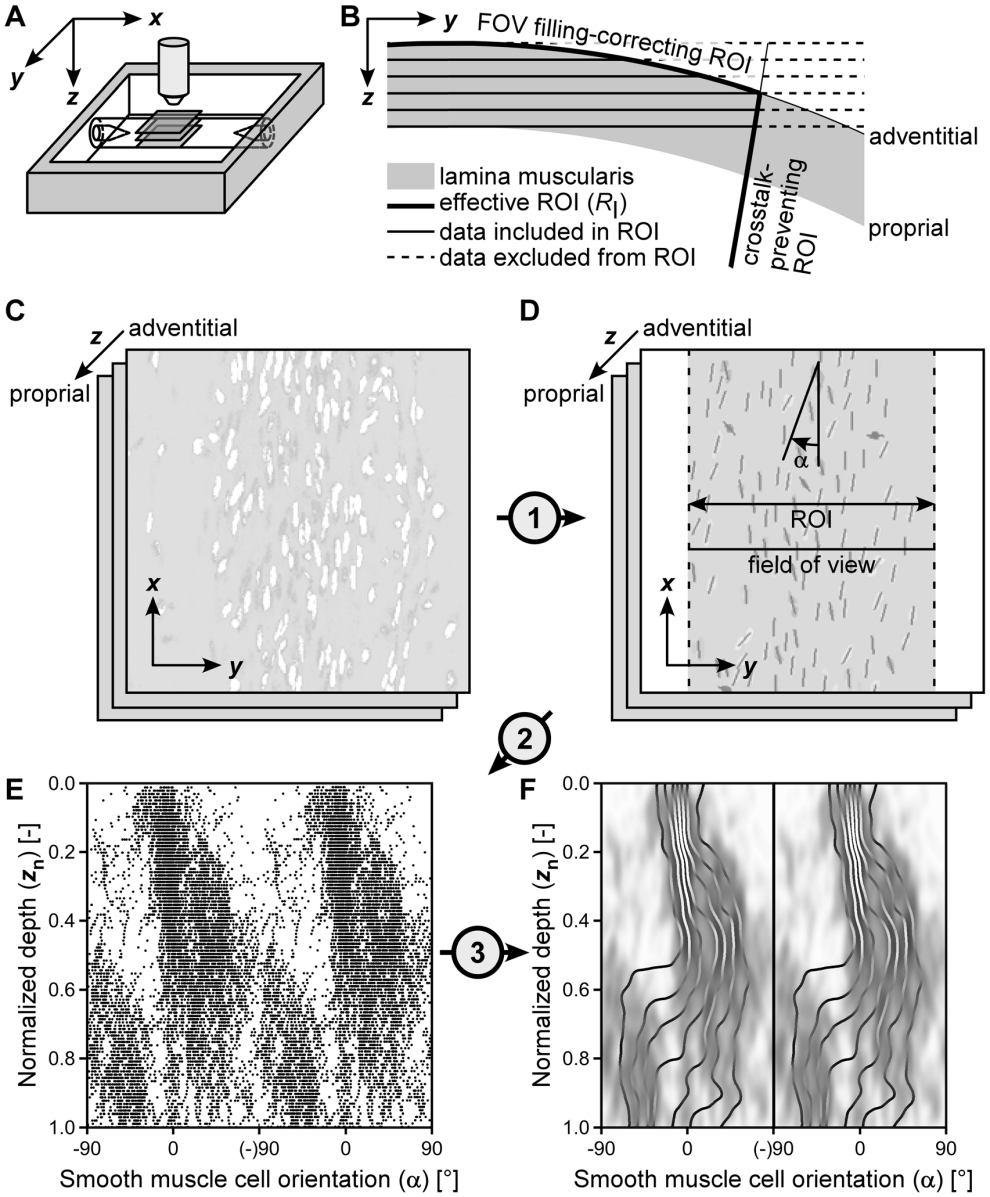
Image acquisition and processing workflow. (**A**) Image stacks of the muscle layer (of the mounted, submerged ureter) were acquired at increasing depth (*z*) from the adventitial to the proprial side, at proximal, middle and distal locations (i.e., three segments per ureter). (**B**) Given the curvature of the vessel, the region of interest (ROI) for quantitative analysis was adjusted to ensure reliable cell density estimation and to limit cross-talk of cells resident at other depths within the wall. (**C**) A stack of raw images, showing smooth muscle cell (SMC) nuclei stained with SYTO 13. (**D**) Raw images (panel **C**) were filtered using cellness filtering (step 1) to identify SMC nuclei. Subsequently, a ROI was applied and individual SMC angles (α) were determined. α was defined with reference to the longitudinal axis of the vessel (*x*-direction). (**E**) SMC angles were plotted as a function of depth to evaluate transmural changes in orientation, taking circularity of the data into account. (**F**) A kernel density estimation (KDE) plot was used to estimate the orientation distribution. On this KDE, octile lines were plotted to clarify changes in orientation dispersion with depth.

Left and right ureters were excised from six Wistar rats, euthanized with CO_2_. After removal of excessive fat, ureters were mounted between glass micropipettes and stained both intra- and extraluminally using 2 µM SYTO 13 (staining cell nuclei) in Hanks' Balanced Salt Solution (HBSS) for 30 minutes (**Figure 1A**). Mounted ureters were imaged using a two-photon laser scanning microscope, acquiring a 3D stack of images traversing the ureter wall from out- to inside at an equal lateral and axial (i.e., in depth or *z* direction) resolution of 0.5 µm. Image stacks were acquired at proximal, middle and distal locations along each ureter, yielding a total of 36 stacks.

### Processing and analysis

In summary, after assessment of ureter diameter, smooth muscle cell nuclei and their orientations were automatically identified in the acquired image stack. For each stack, a depth-dependent region of interest (ROI; see **Figure 1B**) was calculated. This ROI was applied after delineation of SMC nuclei. SMC orientation was only calculated for nuclei within the ROI.

#### Cellness filtering and smooth muscle cell identification

Stack images were filtered using *cellness* filtering, in analogy to vesselness filtering [26]. Cellness filtering enhances elongated structures, e.g., SMC nuclei, in an image, and suppresses background noise, thereby resulting in a high-contrast image with clearly delineated cell nuclei. Cellness-filtered images were converted to binary images by means of thresholding. Pixels of value 1 were clustered and subsequently filtered based on their surface area, removing dye particles (area too small) or adjacent cells that are erroneously clustered as one (area too large). Each cluster now represents a nucleus and signifies an SMC.

#### Region of interest application

As *flat* image slices were acquired of a *curved* object, structures (clusters) at various depths of the wall could end up in one image slice, cf. **Figure 1B**. This crosstalk among slices is reduced by narrowing the region of interest (ROI) used for quantification of SMC orientation (**Figure 1B**). With increasing imaging depth, and with a decreasing radius of curvature of the ureter wall, the ROI is narrowed. Furthermore, for the outermost slices, the imaging field of view is not fully filled with the lamina muscularis (**Figure 1B**). By assuming a cylindrically shaped ureter with a measured radius, the ROI can be chosen such that it is always completely filled with lamina muscularis, allowing cell densities to be calculated.

#### Angle calculation

Nuclear shape was assessed based on the eigenvalues of the structure tensor that was calculated for each identified cluster [27]. The ratio of its eigenvalues is required to be larger than 1.5 to exclude cells with round-shaped nuclei. Principle cluster orientation for the included nuclei is represented by the eigenvector corresponding to the largest eigenvalue. SMC nuclear orientation is expressed by the angle α of this eigenvector with respect to the longitudinal ureter axis (**Figures 1C–E**).

#### Probability density estimation

For each stack, imaging depth was normalized from 0 to 1, after which a two-dimensional kernel density estimate (KDE) [28] was calculated (**Figure 1F**). Kernel density estimation allows for estimation and visualization of the probability density from a set of data points. In our case, the KDE shows how often a certain SMC orientation is encountered at a certain imaging depth. In addition to the KDEs per stack, an overall KDE was calculated of all 36 imaged stacks. For each depth, eight quantiles (octiles) were calculated and displayed as lines on the KDE (**Figure 1F**).

#### Cell density estimation

Cell density (having the unit ‘cells per cross-sectional area’) is calculated by dividing the number of detected nuclei in a slice by the area of the region of interest for that slice. The key difference between cell densities and the aforementioned probability densities is that cell densities are corrected for the ROI and, thus, give a measure of the physical cell density.

For details on imaging and image analysis procedures and calculations, including parameter values, please see the Detailed materials and methods section (**Text S1**) and the Parameter values used in image analysis table (**Table S1**).

## Results

**Figure 2:**
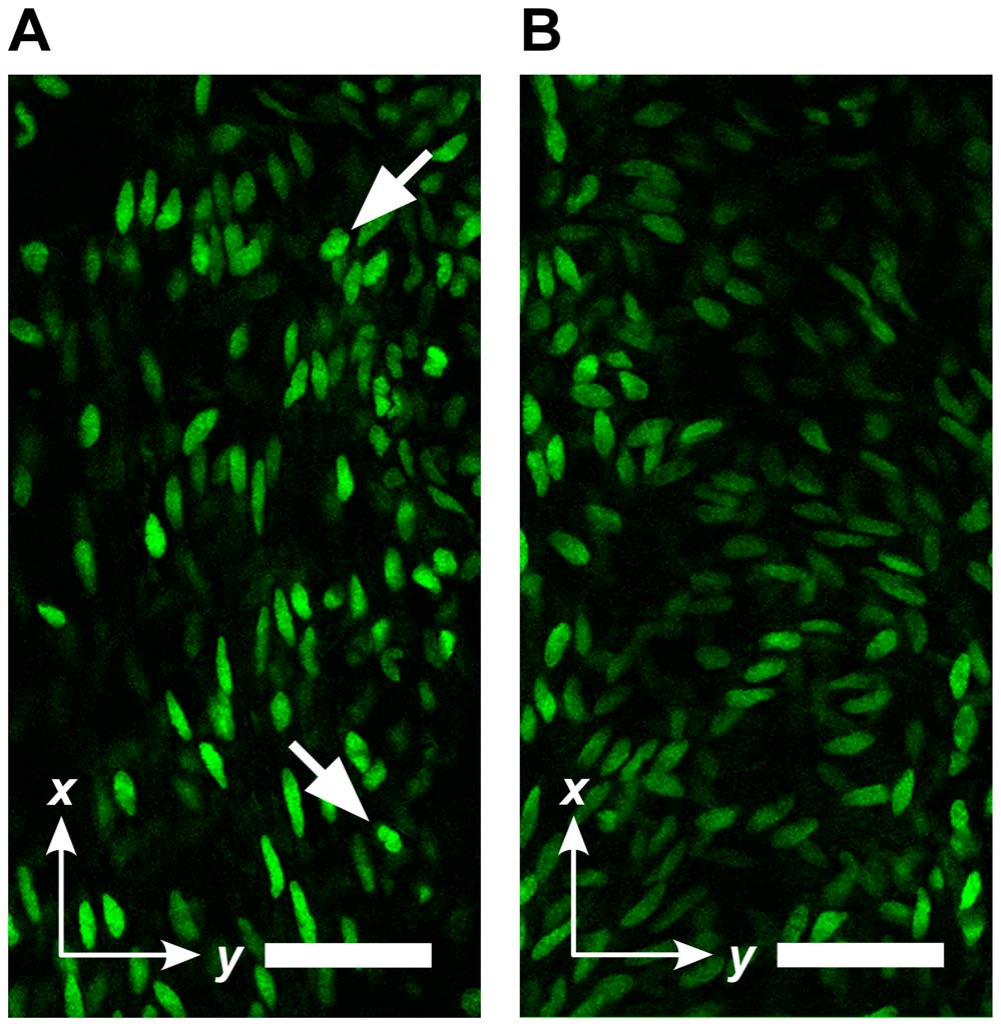
Example image slices from a single image stack. (**A**) Image slice towards the adventitial side, showing predominantly longitudinally oriented smooth muscle cells (SMCs). (**B**) Image slice towards the proprial side, showing a more disperse orientation. Scale bar: 50 µm. Arrows indicate relatively round cells which are excluded from further analysis.

By visual inspection, the acquired TPLSM image stacks typically showed an outer, longitudinal layer of SMCs (**Figure 2A**). With increasing imaging depth, SMC orientation generally dispersed but remained longitudinal (e.g., **Figure 2B**).

In the analysis of SMC orientation, we considered a normalized imaging depth to be able to compare LMs of different thicknesses. The unnormalized median (25–75th percentile) thickness of the 36 SMC layers was 53 (41–70) µm.

**Figure 3:**
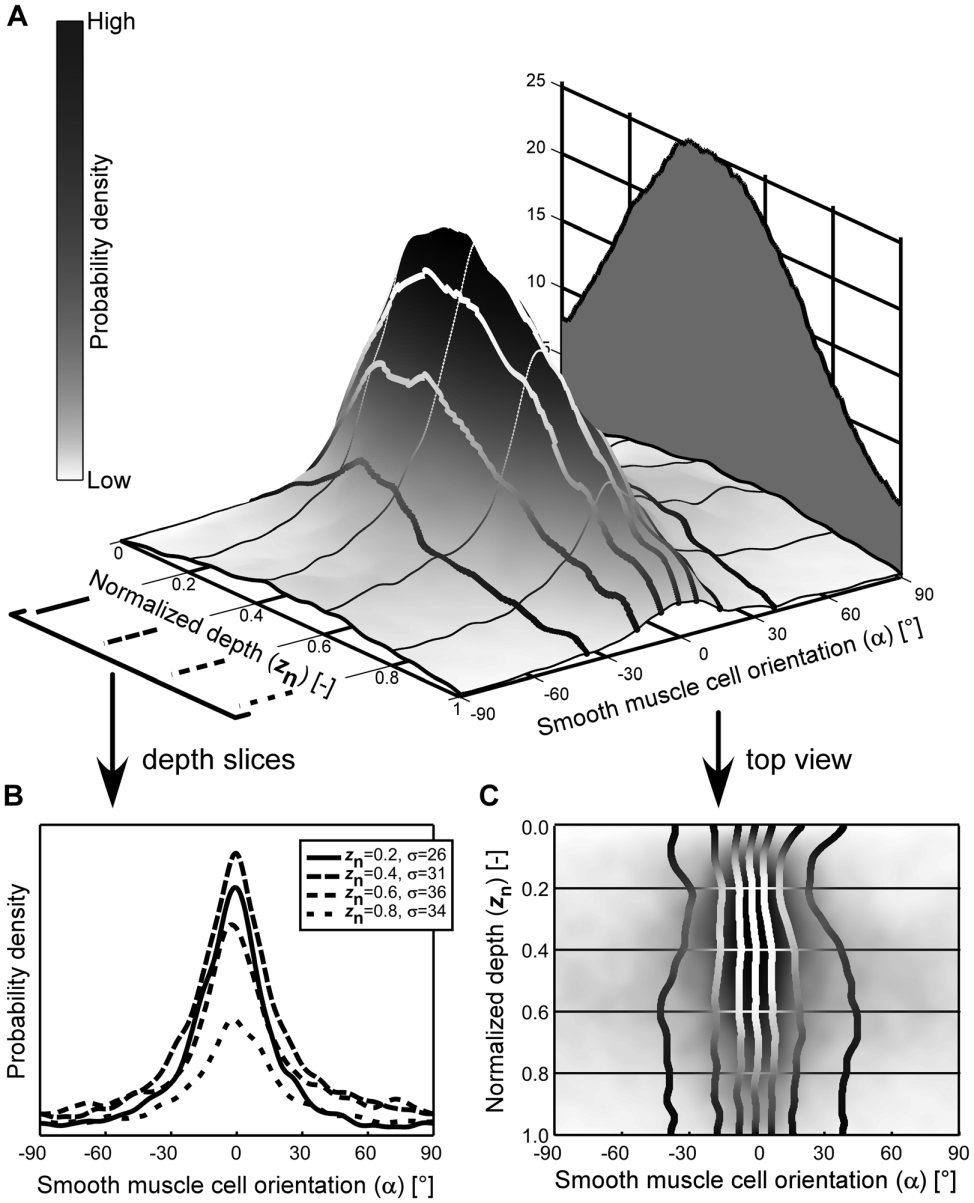
Smooth muscle cell (SMC) orientation in intact rat ureter is predominantly longitudinal. Panel **A** shows the orientation distribution (3D plot) and the cell density (2D graph) as function of depth, averaged over 36 rat ureter segments. Normalized depth 0 corresponds to the adventitial side of the muscle layer and 1 to the proprial side. At the adventitial side (normalized depth 0 to 0.5) there is a high probability that the angle of the SMCs with respect to the longitudinal axis of the ureter is about 0°. Towards the proprial side the SMC orientation gradually disperses but remains centered around 0°, as further illustrated in panel **B** by the distributions at four distinct normalized depths (*z*_n_) as indicated. At these depths, standard deviations (σ) are given. In the distribution plot (**A**) and its top view (**C**) the curves delimit the octiles of the orientation distribution.

Based on our image analysis methodology (concise materials and methods), we quantified SMC orientation, and indeed found a predominantly longitudinal orientation in the 36 rat ureter segments we studied (**Figure 3**). From the adventitial side toward the proprial side we found a clear, but gradual transition from a narrowly longitudinal to a more dispersely longitudinal distribution (**Figures 3A and 3C**). The broadening of the orientation distribution is also evident from its increasing standard deviation with depth (**Figure 3B**). Average SMC density was between 8 cells per 10^4^ µm^2^ (adventitial and proprial sides) and 25 cells per 10^4^ µm^2^ (middle of the LM, **Figure 3A**).

**Figure 4:**
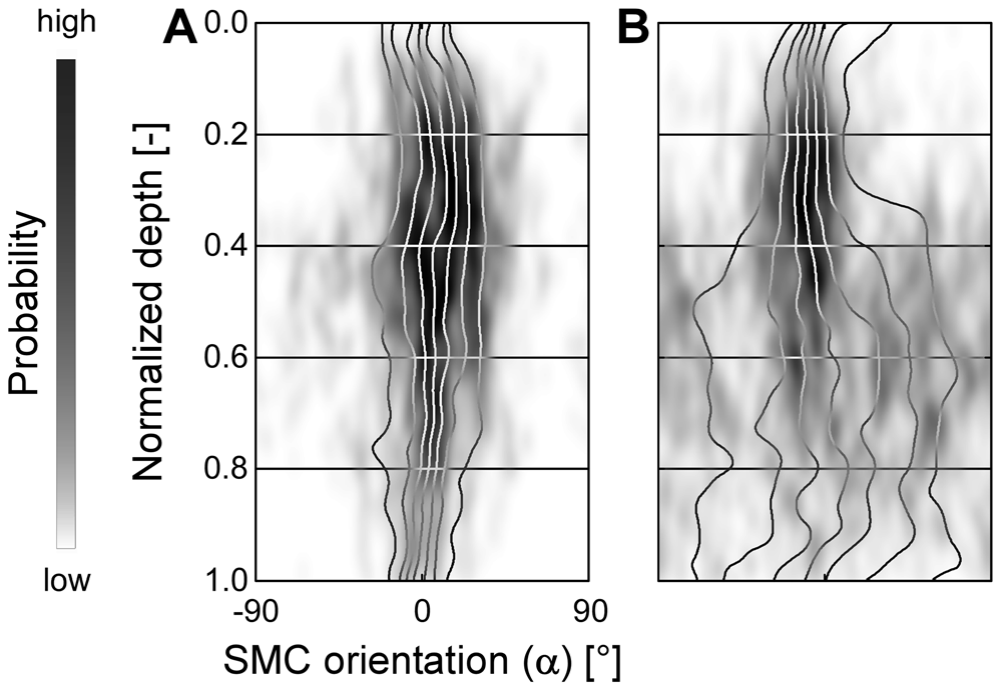
Example orientation patterns of two ureter segments. Panels **A** and **B** (view as defined in **Figure 3C**) show two examples of orientation patterns acquired in different ureter segments. The pattern in **A** remains longitudal from adventitial to proprial side, whereas the pattern in **B** disperses towards the proprial side.

Whereas the main SMC orientation was uniformly longitudinal, individual ureter segments showed slight variability with respect to their dispersion (**Figure 4**). The 50th percentile of the orientation standard deviation (SD) increased from 25° (adventitially) to 32° (proprially) (**Table 1**). Towards the proprial side (normalized depth 0.8), the SD showed a doubled variation among stacks (inter-quartile range (IQR) of 41°-25° = 16°) than at the adventitial side (normalized depth 0.2, IQR of 29°-21° = 8°) (**Table 1**).

**Table 1:** Variability in dispersion among different stacks.

Normalized depth	Percentile of segmental SDs at a given normalized depth		
	25th	50th	75th
**0.2**	21°	25°	29°
**0.4**	24°	29°	37°
**0.6**	25°	32°	48°
**0.8**	25°	32°	41°

To assess variability in dispersion between stacks, we calculated for each stack the orientation standard deviation (SD) at normalized depths 0.2, 0.4, 0.6 and 0.8, as we already did for the entire distribution in **Figure 3B**. The 25th, 50th and 75th percentile of the SD values at each of these normalized depths are shown. Towards the proprial side (normalized depth 0.8), the SD showed a doubled variation among stacks (inter-quartile range (IQR) of 41°-25° = 16°) than at the adventitial side (normalized depth 0.2, IQR of 29°-21° = 8°). Overall, observations between samples consistently show a longitudinal orientation.

## Discussion

The present results, derived from our intact ureter high-resolution imaging technique, show that the LM of the ureter in rat is a single layer with predominantly longitudinally oriented SMCs. Although the dispersion of orientation did vary among individual segments, we found no particular differences between left and right ureters, nor between proximal, middle and distal sites. Since image analysis was fully automated, the quantitative differences among the ureter segments we studied are observer-independent.

The disparity among previous reports on LM structure with regard to SMC orientation and layering may have arisen because of insufficient sampling in the depth direction, apart from the potentially deleterious effects of sectioning. Unlike conventional histological approaches, our high-resolution data was obtained in intact ureters by using TPLSM, an imaging technique similar to confocal laser scanning microscopy (CLSM) [29]. In both techniques, a microscopic sample is scanned point-to-point by a focused laser beam. However, whereas in CLSM, a pinhole is used to accomplish optical sectioning, in TPLSM, optical sectioning is accomplished by the two-photon effect [30]–[32]. As this effect only occurs in at very high light intensities, it only occurs at the laser's focus, and therefore intrinsically leads to optical sectioning [33]. The fact that no pinhole is required greatly increases detection sensitivity. Another advantage of TPLSM when compared to CLSM is the use of long-wavelength laser light, increasing penetration depth [34] and limiting out-of-focus photobleaching. TPLSM has been applied to a wide variety of biological samples [32]. With our co-workers, we have used TPLSM to study the structure of large arteries in mice [35], [36]. Because a rat ureter has approximately the same dimensions as these arteries, TPLSM is an ideal imaging modality to study the ultrastructure of these vessels.

By using TPLSM, we could show that SMC orientation dispersion changes *gradually* from the adventitial to proprial side. If we would have assessed SMC orientation at a limited number of depths across the LM, we could have (erroneously) identified the LM as a layered structure of a highly longitudinal outer layer and dispersely longitudinal inner layer. Unfortunately, most (older) studies on ureter wall and LM structure are rather narrative, lacking quantitative description, and as such may suffer from this pitfall [2]–[8], [10]–[18], [20]–[25].

The predominant longitudinal orientation of SMCs in the LM requires some discussion when peristaltic function is considered. Because shortening of SMCs occurs along their *long* axis [37], our results suggest that SMC contraction would rather locally *shorten* the ureter than cause luminal constriction. However, the role of the ureter wall matrix cannot be neglected. If the latter has a net-like structure throughout the wall, then shortening along the longitudinal axis in one segment will cause luminal constriction in a neighboring segment, in analogy to e.g., a Chinese finger trap [38]. This concept of peristaltic bolus propulsion driven by longitudinally oriented SMCs is corroborated by a study of bolus propulsion in cat esophagus [39], showing that bolus propulsion may be driven by the (coordinated) contraction of SMCs in a neighboring esophageal wall segment. Further support for an active contribution to peristaltic function of longitudinally oriented SMC in the LM of the ureter is provided by the modeling study by Brasseur et al. [1] who studied quantitatively the augmenting effect of longitudinal shortening on luminal constriction of the esophagus as caused primarily by contraction of circumferentially oriented SMCs. Taken together, our findings are not in conflict with the mechanics of peristalsis.

**Figure 5:**
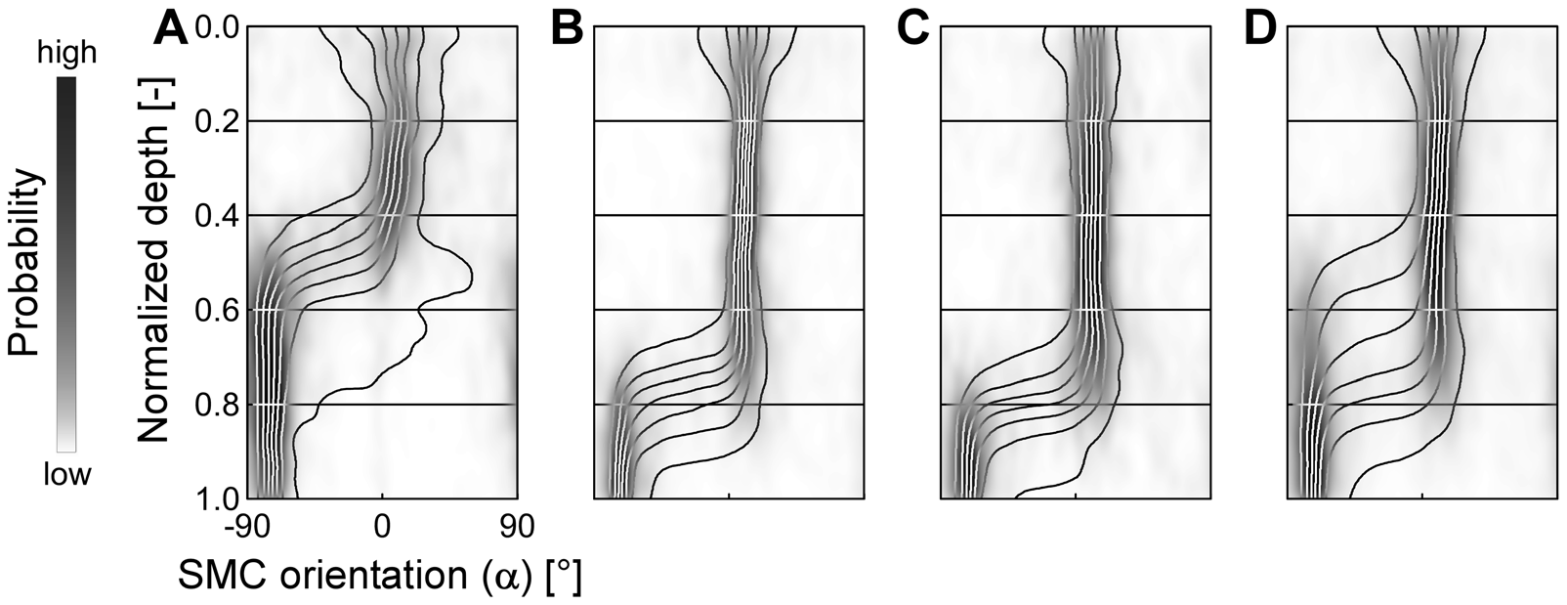
Control sample: By orientation distinguishable smooth muscle layers in the small intestine. In order to verify our image processing method, we applied the exact same preparation and staining method to a rat small intestine, an organ with clearly delineated longitudinal and circumferential smooth muscle layers [8], [10]. Panels **A–C** show orientation distributions analogous to the panels in **Figure 4**, at three different sites in the intestine. Panel **D** shows the average orientation distribution, calculated from panels **A–C**. All panels clearly show a transition from a superficial, longitudinal smooth muscle orientation to a circumferential orientation at the deep end. The patterns shown are all slightly shifted to the right by ∼10°, which is caused by the fact that the two pipettes used to mount the intestine were not perfectly aligned (i.e., microscopy images were rotated by ∼10°).

In order to validate our observation of a *gradually* dispersing SMC orientation across the ureter LM, in a pilot experiment, we imaged a rat small intestine, an organ known to possess clearly *separated* SMC layers [8], [10]. In this organ, we indeed found distinct changes in orientation with depth (**Figure 5**), confirming that the more gradual orientation change we observed in the ureters is unlikely caused by our methodology or by measurement artifacts.

Although our image acquisition and analysis approach provides compelling insight into SMC orientation in the LM of rat ureter, a number of methodological aspects should be considered. We assumed the SMC nucleus orientation to be representative of the orientation of its containing cell body, an assumption also made by Walmsley and Canham [40] and Holzapfel et al. [41]. In an image, the cell *nuclei* appear separated in contrast to than the tightly packed smooth muscle *cells* and, therefore, are easier to delineate. Though Todd et al. state that nuclear orientation is *not* representative of cell orientation [42], it should be kept in mind that other studies describe the SMC to be clearly elongated [43], whereas Todd et al. found cell shape to be irregular; a finding that could be indicative of artifacts due to histological sectioning and mechanical unloading.

A second point of attention is that, using SYTO 13 as a dye, we stained the nuclei of *all* cells in the ureter. By including only anisotropic nuclei in our analyses, we assumed to have assessed only SMCs. In order to verify this assumption, we performed an SMC-specific staining. From previous experiments, we know that staining entire organs (e.g., an artery) specifically for SMCs is very difficult due to the limited penetration depth of available dyes. Therefore, we chose to perform SMC-specific staining (alpha smooth muscle actin) and bright field microscopy in one histologically fixed and sectioned ureter. In the acquired images (**Figure S1**), the adventitia, LM, lamina propria and urothelium are clearly visible. As the adventitia contains very few nuclei, possible crosstalk from non-SMC nuclei in the adventitia is limited. The urothelium, on the other hand, does contain large numbers of nuclei. Because these nuclei are isotropic, they are filtered out and therefore do not influence our quantification.

It is known that in the lamina propria, a layer of connective tissue, fibroblasts are present. Because of the aspecific nuclear dye that we used, also labeling fibroblast nuclei, our scans do not allow identification of the transition from LM to lamina propria. Therefore, with increasing imaging depth, the probability that we are imaging the lamina propria increases, and, hence, we cannot rule out a contribution of fibroblasts to the orientation distribution at the proprial side. This artifact may be partly responsible for the increased dispersion we observed towards this side. However, our data clearly shows that, despite this limitation, the predominant orientation is longitudinal.

**Figure S1** shows that the LM also contains small blood vessels with SMCs. These SMCs could potentially influence our quantification of orientation. Therefore, we took care to acquire TPLSM stacks at such sites that all blood vessels were out of view.

Another methodological aspect to consider is that in our quantitative analysis of each image slice, when calculating the region of interest (ROI), we assumed the ureter wall to be perfectly round. However, because the ureters were not pressurized, their cross-section may have been slightly oval instead of circular. If this were the case, effective radius of curvature would have been larger and potential crosstalk among layers would have been less. Furthermore, the filling-corrected ROI function used is too conservative in case of oval ureters. This potentially removes nuclei of interest and, hence, decreases the signal-to-noise ratio.

Although our method provides quantitative data on (depth-dependent differences in) SMC orientation within the LM, application of our 2D imaging approach to a 3D cylindrical structure has its limitations. First, absolute SMC density is not quantifiable while cell nuclei appear in images at multiple depths (slice interspacing<nucleus dimensions). Second, transverse orientation (i.e., an SMC orientation not fully parallel to the ureter wall) is not measurable while transversely aligned nuclei appear foreshortened (i.e., round shaped) in the image, which may lead to elimination in the cellness filtering process (based on lack of anisotropy). In the future, 3D processing of the image stack could address these issues. For further mechanistic studies on ureter wall structure and peristaltic function, matrix composition and orientation should be further quantified and intra-vital imaging of actual ureter peristalsis should be performed.

In the present study, we imaged ureters of rats. It should be mentioned that although rat ureter structure and function cannot be directly translated to the human situation, our technique does offer an unprecedented view of the *intact* ureter ultrastructure. Presently, no techniques are available to accomplish this level of detail in larger (i.e., human) intact ureters.

From this study, we conclude that smooth muscle cell orientation in rat ureter is predominantly longitudinal. The observed gradual dispersion towards the proprial side does not support identification of separate layers. Our minimally invasive and quantitative image acquisition and analysis approach is a crucial step towards more quantitative insight into ureter peristaltic function, and can also be used to study smooth muscle cell orientation in other tube-like structures, e.g., in gut and in blood vessels.

## Supporting Information

Text S1 **Detailed materials and methods.**
(DOCX)

Table S1
**Parameter values used in image analysis.** Parameters were chosen to obtain optimal results. 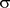 represents the standard deviation and therefore approximately half the width of the Gaussian derivative kernel used in cellness filtering. In order to optimally detect the SMC nuclei, the kernel width should be approximately equal to the width of the SMCs, which, in our case, was about 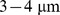. 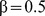 was known to give good results in other studies [26]. 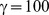 was determined empirically. It should be noted that the choice of 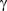 depends on the intensity of the acquired images. The value of 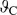 was not critical (cellness rapidly decreases to very low values outside nuclei). 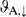 and 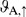 were determined empirically, however, an estimate of the SMC cross-sectional area can be calculated from the SMC nucleus' mean short axis (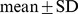, 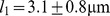 for an aortic SMC [44]) and long axis (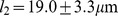 for an aortic SMC [44]) lengths, assuming an elliptically shaped cross-section: 
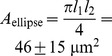
. Therefore, 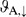 and 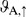 do include SMC nuclei of normal sizes. The choice of 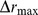 was a tradeoff between minimization of crosstalk (small 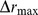) and number of included nuclei (large 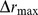). 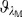 was safely set to 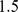 (actual SMC anisotropy (e.g., 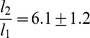 [44]) is much larger). 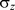 and 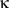 were chosen by assessing probability density estimates calculated using various combinations of 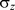 and 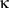 and choosing those values that gave the optimal trade-off between noise and detail.(DOCX)

Figure S1 **Alpha smooth muscle actin staining in histological ureter sections.** Panels A and B show cross-sectional and longitudinal sections of a rat ureter, stained for alpha smooth muscle actin (brown) and nuclei (purple). Scale bar: 50 µm. L, lumen; V, blood vessel. Histology was performed on a ureter fixed in 4% buffered paraformaldehyde, routinely processed, embedded in paraffin, and sectioned at 4 µm. Sections were labeled with monoclonal anti-alpha smooth muscle actin-fluorescein isothiocyanate (FITC) antibodies (Sigma-Aldrich, St. Louis, MO) and successively stained with anti-FITC horseradish peroxidase (HRP). Nuclei were stained using hematoxylin. Imaging was performed using a Nikon Eclipse 800 microscope (Nikon Instruments Inc., Melville, NY) equipped with a Nikon S Fluor 40×/1.30 oil immersion objective. Images were acquired using a Media Cybernetics Evolution VF camera (Media Cybernetics Inc., Rockville, MD).
(DOCX)
