# Supplementary material for: Ureter Smooth Muscle Cell Orientation in Rat Is Predominantly Longitudinal
Source: PLoS One. 2014 Jan 21;9(1):e86207. doi: 10.1371/journal.pone.0086207 (PMC3897663; doi:10.1371/journal.pone.0086207)
Supplement: Text S1 — Detailed materials and methods. (DOCX) [file pone.0086207.s001.docx]

Ureter smooth muscle cell orientation in rat is predominantly longitudinal

Bart Spronck^1^, Jort J. Merken^1^, Koen D. Reesink, Wilco Kroon, and Tammo Delhaas

1. Both authors contributed equally.

# Text S1. Detailed Materials and Methods

## Experimental procedures

Experiments and procedures were approved by the local ethics committee on use of laboratory animals. Our experimental animal group consisted of six Wistar rats, from the experimental animal center of Maastricht University. Animals were euthanized with CO_2_. Within 5 minutes after euthanasia, the ureters were approached via a median incision, opening the abdominal cavity. Proximal and distal ends of the ureter were cut 1 mm from the renal pelvis and 5 mm from the bladder, respectively. Identification of the proximal end was achieved by applying surgical wire to the proximal end. Distinction between left and right ureter samples was achieved by tying one wire on the proximal end of left-sided and two wires on the proximal end of right-sided ureters. Excessive fat surrounding the ureters was manually removed under a stereomicroscope. Next, the excised rat ureters were mounted between glass micropipettes in a custom-built ureter mounting chamber (**Figure 1A**), containing Hanks' Balanced Salt Solution (HBSS; Life Technologies Europe BV, Bleiswijk, the Netherlands) in which the ureter remained immersed during the whole staining and imaging process. The HBSS we used had a pH of 7.4 and contained NaCl (137 mM), HEPES (15 mM), glucose (5.5 mM), KCl (5.3 mM), CaCl_2_ (2.5 mM), Na_2_HPO_4_ (0.34 mM), KH_2_PO_4_ (0.44 mM) and MgSO_4_ (1.1 mM). Specimens were stained with the DNA/RNA fluorescent dye SYTO 13 ($\lambda_{max,emission}=520 \mathrm{nm}$; Life Technologies), diluted with HBSS to a final concentration of 2 µM. HBSS in the mounting chamber was replaced with the diluted SYTO 13, as was the intraluminal HBSS. To avoid bleaching of the staining solution by ambient light, the setup was covered with aluminum foil during staining. After 30 minutes, the staining solution was again replaced with pure HBSS. Imaging was performed with a two-photon laser scanning microscopy (TPLSM) system (Leica TCS SP5 MP, Leica Mikrosysteme Vertrieb GmbH, Wetzlar, Germany) equipped with an HCX APO L 20x/1.00W water dipping objective and a Hamamatsu R9624 (Hamamatsu Photonics, Hamamatsu city, Japan) photomultiplier tube (PMT). Two-photon excitation was achieved using a pulsed Ti-Sapphire laser (Chameleon Ultra II, Coherent Inc., Santa Clara, USA) at a wavelength of 800 nm. Laser power was kept as low as possible to avoid tissue damage. Scanning frequency was set to 600 lines per second. Resolution (1480x1480 pixels) and field of view size (738x738 µm^2^) were kept constant throughout the imaging process, resulting in a pixel size of 0.5x0.5 µm^2^.
Image stacks were acquired at 12 bits and with an inter-slice distance of 0.5 µm, traversing the ureter wall from out- to inside. Three image stacks were acquired along the length of the ureter at proximal, middle and distal positions. After acquisition, lateral field of view was reduced by cropping the images to a resolution of 512x512 pixels to ease further processing and limit computation time.

## Processing and analysis

### Cropping and diameter assessment

In each acquired stack, ureter diameter was assessed by showing a transversal section through the stack and drawing a circle on screen using ImageJ 1.47v (National Institutes of Health, Bethesda, Maryland, USA). After diameter assessment, the lateral field of view was reduced by cropping the images to a resolution of 512x512 pixels to ease further processing and limit computation time. All further processing was performed using MATLAB R2013b (The MathWorks Inc, Natick, Massachusetts, USA).

### Cellness filtering

Stack images were filtered using cellness filtering, in analogy to vesselness filtering [[1](#_ENREF_1)]. This filtering is a technique originally developed to enhance blood vessels in 2D or 3D image stacks [[1](#_ENREF_1)]. Cellness filtering enhances elongated structures (in this case, SMC nuclei) in an image and suppresses background noise, thereby resulting in a high-contrast image with clearly delineated cell nuclei.

First, for each pixel, a Hessian matrix $\mathbb{H}$ is computed:

$\mathbb{H(}x,y)=\left[ \begin{matrix} \left. \frac{\partial^{2}I}{\partial x^{2}} \right|_{x,y} & \left. \frac{\partial^{2}I}{\partial x\partial y} \right|_{x,y} \\ \left. \frac{\partial^{2}I}{\partial x\partial y} \right|_{x,y} & \left. \frac{\partial^{2}I}{\partial y^{2}} \right|_{x,y} \end{matrix} \right]$ ,

with $I(x,y)$ being the image intensity at a given point. Hessian terms are calculated by convolution of the image with the respective second-order derivatives of a 2D Gaussian kernel $G$:

$G\left( x,y \right)=\frac{e^{{-\left( x^{2}+y^{2} \right)}/{2\sigma^{2}}}}{2\pi\sigma^{2}}$ ,

in which $\sigma$ is the kernel standard deviation (**Table S1**). Second, for each pixel, the eigenvalues ($\left| \lambda_{\mathbb{H},1} \right|\leq\left| \lambda_{\mathbb{H},2} \right|$) of $\mathbb{H}$ are computed, which are subsequently used to calculate blobness ($0\leq R_{B}\leq1$) and structureness ($S\geq0$):

$R_{B}= \left| \frac{\lambda_{\mathbb{H},1}}{\lambda_{\mathbb{H},2}} \right|$ , and

$S= \sqrt{\lambda_{\mathbb{H},1}^{2}+\lambda_{\mathbb{H},2}^{2}}$ .

For pixels in an isotropic structure, in which the eigenvalues are approximately equal, $R_{B}=1$; whereas for pixels in an anisotropic structure, $R_{B}\to0$. Cell pixels can be distinguished from background pixels by $S$, because the magnitude of the derivatives (and thus of the eigenvalues) in background pixels is small [[1](#_ENREF_1)]. $R_{B}$ and $S$ are combined into cellness ($0\leq C\leq1$) using

$C=\left\{ \begin{matrix} 0 & \mathrm{if}\lambda_{\mathbb{H},2}>0 \\ \left( e^{-{R_{B}^{2}}/{2\beta^{2}}} \right)\cdot\left( 1-e^{-{S^{2}}/{2\gamma^{2}}} \right) & \end{matrix} \right.$ ,

with $\beta$ and $\gamma$ being parameters weighing blobness and structureness, respectively (**Table S1**).

### Smooth muscle cell identification

Cellness-filtered images were thresholded at $\vartheta_{C}$ (**Table S1**), yielding binary images. In these images, pixels of value 1 were clustered based on their 8-adjacency [[2](#_ENREF_2)], each cluster signifying an SMC. Clusters were subsequently filtered based on the their surface area using lower and upper thresholds of $\vartheta_{A,\downarrow}$ and $\vartheta_{A,\uparrow}$, respectively (**Table S1**).

### Region of interest application

As *flat* image slices were acquired of a *curved* object, structures (clusters) at various depths of the wall could end up in one image slice. This crosstalk among slices can be reduced by narrowing the ROI used for quantification of SMC orientation (**Figure 1B**). With increasing imaging depth ($z$), and thus with a decreasing radius of curvature of the ureter wall, the ROI should narrow. For a certain effective slice thickness (${\Delta r}_{\max}=r_{2}-r_{1}=6 \mu m$, **Figure 1B** and **Table S1**), it can be shown that for the crosstalk-limiting ROI ($R_{I,cl}(z)$), it holds that:

$R_{I,cl}(z)= 2 \cdot\sqrt{\left( r_{0}-z+{\Delta r}_{\max} \right)^{2}-{(r_{0}-z)}^{2}}$ ,

with $r_{0}$ being the outer ureter radius, i.e., the distance from the ureter axis to the outermost muscle layer.

For the outermost slices, the crosstalk-limiting ROI is not fully filled with the lamina muscularis (**Figure 1B**). In order to correctly estimate SMC density, which will be calculated by dividing the number of detected cells within a slice's ROI by the surface area of this ROI, a filling-corrected ROI ($R_{I,fc}(z)$) was defined:

$R_{I,fc}(z)=2\cdot\sqrt{r_{0}^{2}-\left( r_{0}-z \right)^{2}}$ .

For $z=\Delta r_{\max}$,$R_{I,cl}(z)=R_{I,fc}(z)$. From this depth ($z=\Delta r_{\max}$), $R_{I,cl}(z)$ is again used as the normalizing function. In summary, the overall ROI function ($R_{I}(z)$) can be formulated as

$R_{I}\left( z \right)=\left\{ \begin{matrix} 2\cdot\sqrt{r_{0}^{2}-\left( r_{0}-z \right)^{2}} & \mathrm{if}z\leq\Delta r_{\max} \\ 2 \cdot\sqrt{\left( r_{0}-z+{\Delta r}_{\max} \right)^{2}-{(r_{0}-z)}^{2}} & \mathrm{if}z>\Delta r_{\max} \end{matrix} \right.$ .

### Orientation calculation

In order to obtain the principal axes of the clusters of interest (i.e., clusters that satisfy the area criteria $\vartheta_{A,\downarrow}$ and $\vartheta_{A,\uparrow}$, and that are within the ROI), for each of these clusters, a structure tensor ($\mathbb{M}$) was calculated [[3](#_ENREF_3),[4](#_ENREF_4)]:

$\mathbb{M=}\left[ \begin{matrix} \sum\left( x-x_{c} \right)^{2} & \sum\left( x-x_{c} \right)\left( y-y_{c} \right) \\ \sum\left( x-x_{c} \right)\left( y-y_{c} \right) & \sum\left( y-y_{c} \right)^{2} \end{matrix} \right]$ ,

with $(x_{c},y_{c})$ the cluster's center of mass. Nuclear shape can now be assessed based on the eigenvalues ($\lambda_{\mathbb{M},1}\leq\lambda_{\mathbb{M},2}$) of $\mathbb{M}$. Only clearly elongated clusters (${\lambda_{\mathbb{M},2}}/{\lambda_{\mathbb{M},1}}\geq\vartheta_{\lambda_{\mathbb{M}}}$, **Table S1**) were considered in the analysis to include SMC nuclei but to exclude e.g., fibroblasts. For the included nuclei, the eigenvector corresponding to the largest eigenvalue ($\lambda_{\mathbb{M},2}$) of $\mathbb{M}$ represents the principal cluster orientation. Nuclear orientation is expressed by the angle $\alpha$ of this eigenvector with the longitudinal ureter axis (**Figures 1C-E**).

### Probability density estimation

For each stack, $z$ was normalized to a range of $\left[ 0, 1 \right]$, yielding $z_{n}$. Subsequently, a two-dimensional ($\alpha,z_{n}$) kernel density estimate (KDE [[5](#_ENREF_5)]) was calculated^^[[1]](#footnote-1)^^ (**Figure 1F**) [[6](#_ENREF_6)]. Kernel density estimation allows for estimation and visualization of the probability density from a set of data points, which, in our case, shows how often a certain SMC orientation is observed at a certain imaging depth. Briefly, the contribution of each data point ($\alpha_{c},z_{n,c}$) is spread out over a (relatively small) area [[6](#_ENREF_6),[7](#_ENREF_7)]; the KDE at a given depth ($z_{n}$) in a given direction ($\alpha$) is then the sum of the contributions of the smeared-out points at this ($\alpha,z_{n}$), and equals 1 when integrated over the full ($\alpha,z_{n}$)-plane. The contributions are described by a two-dimensional function, the so-called 'kernel'. In depth direction, this kernel was normally shaped, whereas in the angular direction, a Von-Mises shape is used:

$K\left( \alpha,z_{n} \right)=\frac{1}{N}\frac{e^{{-\left( z_{n}-z_{n,c} \right)^{2}}/{2{\sigma_{z}}^{2}}}}{\sqrt{2\pi}\sigma_{z}}\frac{e^{\kappa\cos\left( 2\left( \alpha-\alpha_{c} \right) \right)}}{2\pi I_{0}\left( \kappa\right)}$ ,

where $N$ is the total number of data points per ureter segment, ($\alpha_{c},z_{n,c}$) is the kernel center, $\sigma_{z}$ is the kernel width in $z$-direction, $\kappa$ is a measure of concentration in angular direction, and $I_{0}$ is a modified Bessel function of order 0. The at first sight unusual choice of a Von-Mises distribution is required since alpha describes a direction (ranging from, e.g., -90 to 90 degrees) [[7](#_ENREF_7)]. A kernel in $\alpha$-direction, therefore, must be $\pi$-periodic. $\sigma_{z}$ and $\kappa$ were set to $0.02$ and $100$, respectively (**Table S1**).

In addition to the KDEs per ureter segment, an overall KDE was calculated of all 36 imaged ureters by essentially adding all 36 separate KDEs and dividing by 36.

For each depth, eight quantiles (octiles) were calculated and displayed as lines on the KDE^^[[2]](#footnote-2)^^.

### Cell density estimation

Cell density at a given slice ($z$), having the unit 'cells per cross-sectional area', is calculated by dividing the number of detected nuclei in this slice by the surface area of the region of interest $R_{I}(z)$ for that slice. The key difference between cell densities and the aforementioned probability densities is that cell densities are corrected for the ROI and, thus, give a measure of the physical cell density.

# REFERENCES

1. Frangi AF, Niessen WJ, Vincken KL, Viergever MA (1998) Multiscale vessel enhancement filtering. Medical Image Computing and Computer-Assisted Intervention - Miccai'98 1496: 130-137.

2. Gonzalez RC, Woods RE (2008) Digital image processing. Upper Saddle River, N.J.: Prentice Hall. 954 p.

3. Jähne B (1993) Spatio-temporal Image Processing; Goos GH, Juris, editor. Berlin Heidelberg: Sprinter-Verlag. 208 p.

4. Vader D, Kabla A, Weitz D, Mahadevan L (2009) Strain-induced alignment in collagen gels. PloS one 4: e5902.

5. Silverman BW (1986) Density estimation for statistics and data analysis. London; New York: Chapman and Hall. 175 p.

6. Fisher NI (1989) Smoothing a sample of circular data. Journal of Structural Geology 11: 775-778.

7. Fisher NI (1993) Statistical analysis of circular data. New York, NY, USA: Cambridge University Press. 296 p.

1. Kernel density estimation is comparable to the calculation of a histogram, but, has an important advantage, since the mandatory choice of origin of the histogram (and, therefore, of the cutoff values for each bin, potentially influencing results) is avoided. [↑](#footnote-ref-1)
2. Because of data circularity, the middle octile line does *not* represent the median angle. [↑](#footnote-ref-2)
