# Supplementary material for: Ureter Smooth Muscle Cell Orientation in Rat Is Predominantly Longitudinal
Source: PLoS One. 2014 Jan 21;9(1):e86207. doi: 10.1371/journal.pone.0086207 (PMC3897663; doi:10.1371/journal.pone.0086207)
Supplement: Table S1 — Parameter values used in image analysis. Parameters were chosen to obtain optimal results. represents the standard deviation and therefore approximately half the width of the Gaussian derivative kernel used in cellness filtering. In order to optimally detect the SMC nuclei, the kernel width should be approximately equal to the width of the SMCs, which, in our case, was about . was known to give good results in other studies [26]. was determined empirically. It should be noted that the choice of depends on the intensity of the acquired images. The value of was not critical (cellness rapidly decreases to very low values outside nuclei). and were determined empirically, however, an estimate of the SMC cross-sectional area can be calculated from the SMC nucleus' mean short axis (, for an aortic SMC [44]) and long axis ( for an aortic SMC [44]) lengths, assuming an elliptically shaped cross-section: . Therefore, and do include SMC nuclei of normal sizes. The choice of was a tradeoff between minimization of crosstalk (small ) and number of included nuclei (large ). was safely set to (actual SMC anisotropy (e.g., [44]) is much larger). and were chosen by assessing probability density estimates calculated using various combinations of and and choosing those values that gave the optimal trade-off between noise and detail. (DOCX) [file pone.0086207.s002.docx]

Ureter smooth muscle cell orientation in rat is predominantly longitudinal

Bart Spronck^1^, Jort J. Merken^1^, Koen D. Reesink, Wilco Kroon, and Tammo Delhaas

1. Both authors contributed equally.

**Table S1.** Parameter values used in image analysis

| Symbol | Value | Unit | Description |
| --- | --- | --- | --- |
| $\sigma$ | 1.9 | µm | Cellness filtering kernel width (equals 4 pixels). |
| $\beta$ | 0.5 | - | Cellness filtering parameter weighing blobness, which is used to distinguish blob-like from line-like structures. |
| $\gamma$ | 100 | - | Cellness filtering parameter weighing image intensity. |
| $\vartheta_{C}$ | 0.01 | - | Threshold used to convert cellness images to binary images. |
| $\vartheta_{A,\downarrow}$ | 23 | µm^2^ | Lower cross-sectional area threshold (equals 100 pixels). Clusters with a smaller cross-sectional area than $\vartheta_{A,\downarrow}$ are filtered out. |
| $\vartheta_{A,\uparrow}$ | 230 | µm^2^ | Upper cross-sectional area threshold (equals 1000 pixels). Clusters with a larger cross-sectional area than $\vartheta_{A,\uparrow}$ are filtered out. |
| $\Delta r_{\max}$ | 6 | µm | Effective slice thickness, i.e., the maximum crosstalk depth. |
| $\vartheta_{\lambda_{\mathbb{M}}}$ | 1.5 | - | Lower threshold for the ratio of eigenvalues (${\lambda_{\mathbb{M},2}}/{\lambda_{\mathbb{M},1}};$i.e., anisotropy) of a cluster. Clusters with a ratio below this value are filtered out. |
| $\sigma_{z}$ | 0.02 | - | Probability density estimation kernel size in depth-direction. |
| $\kappa$ | 100 | - | Probability density estimation kernel concentration parameter in angular-direction. |

Parameters were chosen to obtain optimal results. $\sigma$ represents the standard deviation and therefore approximately half the width of the Gaussian derivative kernel used in cellness filtering. In order to optimally detect the SMC nuclei, the kernel width should be approximately equal to the width of the SMCs, which, in our case, was about $3-4 \mu m$. $\beta=0.5$ was known to give good results in other studies [[1](#_ENREF_1)]. $\gamma=100$ was determined empirically. It should be noted that the choice of $\gamma$ depends on the intensity of the acquired images. The value of $\vartheta_{C}$ was not critical (cellness rapidly decreases to very low values outside nuclei). $\vartheta_{A,\downarrow}$ and $\vartheta_{A,\uparrow}$ were determined empirically, however, an estimate of the SMC cross-sectional area can be calculated from the SMC nucleus' mean short axis ($mean\pm SD$, $l_{1}=3.1\pm0.8 \mu m$ for an aortic SMC [[2](#_ENREF_2)]) and long axis ($l_{2}=19.0\pm3.3 \mu m$ for an aortic SMC [[2](#_ENREF_2)]) lengths, assuming an elliptically shaped cross-section: $A_{\mathrm{ellipse}}=\frac{\pi l_{1}l_{2}}{4}=46\pm15 {\mu m}^{2}$. Therefore, $\vartheta_{A,\downarrow}$ and $\vartheta_{A,\uparrow}$ do include SMC nuclei of normal sizes. The choice of $\Delta r_{\max}$ was a tradeoff between minimization of crosstalk (small $\Delta r_{\max}$) and number of included nuclei (large $\Delta r_{\max}$). $\vartheta_{\lambda_{\mathbb{M}}}$ was safely set to $1.5$ (actual SMC anisotropy (e.g., $\frac{l_{2}}{l_{1}}=6.1\pm1.2$ [[2](#_ENREF_2)]) is much larger). $\sigma_{z}$ and $\kappa$ were chosen by assessing probability density estimates calculated using various combinations of $\sigma_{z}$ and $\kappa$ and choosing those values that gave the optimal trade-off between noise and detail.

# References

1. Frangi AF, Niessen WJ, Vincken KL, Viergever MA (1998) Multiscale vessel enhancement filtering. Medical Image Computing and Computer-Assisted Intervention - Miccai'98 1496: 130-137.

2. O'Connell MK, Murthy S, Phan S, Xu C, Buchanan J, et al. (2008) The three-dimensional micro- and nanostructure of the aortic medial lamellar unit measured using 3D confocal and electron microscopy imaging. Matrix biology : journal of the International Society for Matrix Biology 27: 171-181.
