# Supplementary material for: Ureter Smooth Muscle Cell Orientation in Rat Is Predominantly Longitudinal
Source: PLoS One. 2014 Jan 21;9(1):e86207. doi: 10.1371/journal.pone.0086207 (PMC3897663; doi:10.1371/journal.pone.0086207)
Supplement: Figure S1 — Alpha smooth muscle actin staining in histological ureter sections. Panels A and B show cross-sectional and longitudinal sections of a rat ureter, stained for alpha smooth muscle actin (brown) and nuclei (purple). Scale bar: 50 µm. L, lumen; V, blood vessel. Histology was performed on a ureter fixed in 4% buffered paraformaldehyde, routinely processed, embedded in paraffin, and sectioned at 4 µm. Sections were labeled with monoclonal anti-alpha smooth muscle actin-fluorescein isothiocyanate (FITC) antibodies (Sigma-Aldrich, St. Louis, MO) and successively stained with anti-FITC horseradish peroxidase (HRP). Nuclei were stained using hematoxylin. Imaging was performed using a Nikon Eclipse 800 microscope (Nikon Instruments Inc., Melville, NY) equipped with a Nikon S Fluor 40×/1.30 oil immersion objective. Images were acquired using a Media Cybernetics Evolution VF camera (Media Cybernetics Inc., Rockville, MD). (DOCX) [file pone.0086207.s003.docx]

Ureter smooth muscle cell orientation in rat is predominantly longitudinal

Bart Spronck^1^, Jort J. Merken^1^, Koen D. Reesink, Wilco Kroon, and Tammo Delhaas

1. Both authors contributed equally.

**
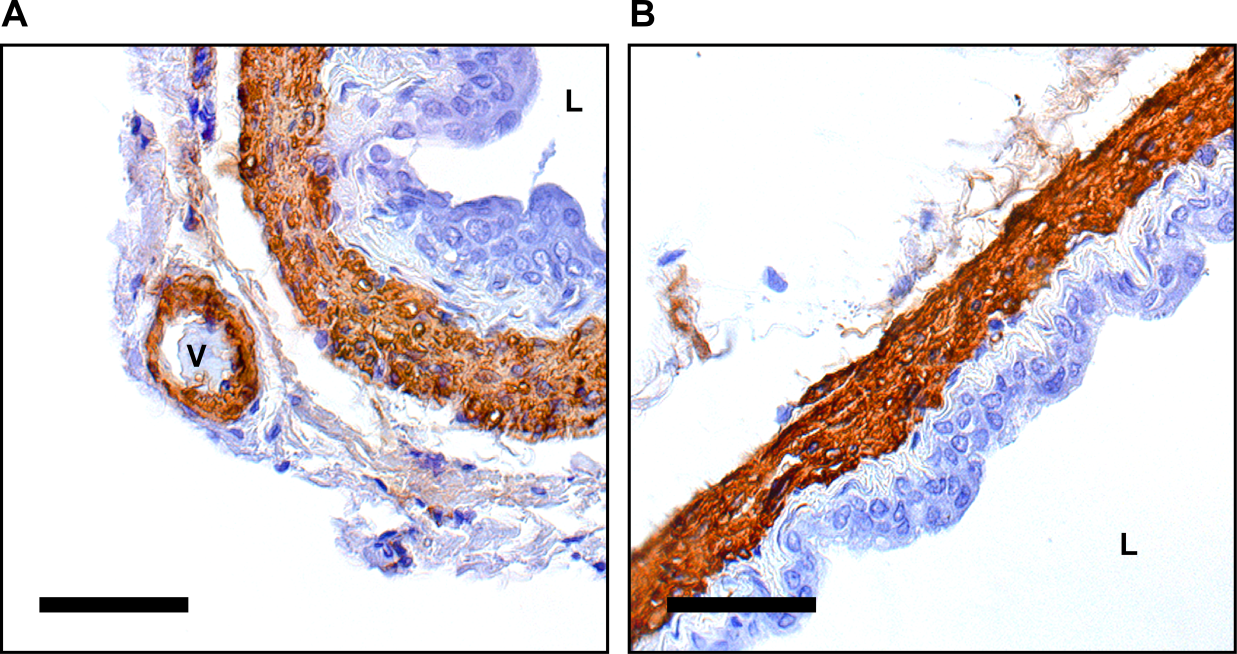
**

**Figure S1. Alpha smooth muscle actin staining in histological ureter sections.** Panels A and B show cross-sectional and longitudinal sections of a rat ureter, stained for alpha smooth muscle actin (brown) and nuclei (purple). Scale bar: 50 µm. L, lumen; V, blood vessel.

Histology was performed on a ureter fixed in 4% buffered paraformaldehyde, routinely processed, embedded in paraffin, and sectioned at 4 μm. Sections were labeled with monoclonal anti-alpha smooth muscle actin-fluorescein isothiocyanate (FITC) antibodies (Sigma-Aldrich, St. Louis, MO) and successively stained with anti-FITC horseradish peroxidase (HRP). Nuclei were stained using hematoxylin. Imaging was performed using a Nikon Eclipse 800 microscope (Nikon Instruments Inc., Melville, NY) equipped with a Nikon S Fluor 40x/1.30 oil immersion objective. Images were acquired using a Media Cybernetics Evolution VF camera (Media Cybernetics Inc., Rockville, MD).
